# Electrical Stimulation over Bilateral Occipito-Temporal Regions Reduces N170 in the Right Hemisphere and the Composite Face Effect

**DOI:** 10.1371/journal.pone.0115772

**Published:** 2014-12-22

**Authors:** Li-Zhuang Yang, Wei Zhang, Bin Shi, Zhiyu Yang, Zhengde Wei, Feng Gu, Jing Zhang, Guanbao Cui, Ying Liu, Yifeng Zhou, Xiaochu Zhang, Hengyi Rao

**Affiliations:** 1 CAS Key Laboratory of Brain Function & Disease, and School of Life Sciences, University of Science and Technology of China, Hefei, Anhui, China; 2 Provincial Hospital Affiliated to Anhui Medical University, Hefei, Anhui, China; 3 The First Affiliated Hospital, Anhui University of Chinese Medicine, Hefei, Anhui, China; 4 Center of Medical Physics and Technology, Hefei Institutes of Physical Science, CAS, Hefei, Anhui, China; 5 School of Humanities & Social Science, University of Science and Technology of China, Hefei, Anhui, China; 6 Center for Functional Neuroimaging, Department of Neurology and Radiology, University of Pennsylvania, Philadelphia, Pennsylvania, United States of America; University of Hong Kong, Hong Kong

## Abstract

Transcranial direct current stimulation (tDCS) is a non-invasive brain stimulation technique that can modulate cortical excitability. Although the clinical value of tDCS has been advocated, the potential of tDCS in cognitive rehabilitation of face processing deficits is less understood. Face processing has been associated with the occipito-temporal cortex (OT). The present study investigated whether face processing in healthy adults can be modulated by applying tDCS over the OT. Experiment 1 investigated whether tDCS can affect N170, a face-sensitive ERP component, with a face orientation judgment task. The N170 in the right hemisphere was reduced in active stimulation conditions compared with the sham stimulation condition for both upright faces and inverted faces. Experiment 2 further demonstrated that tDCS can modulate the composite face effect, a type of holistic processing that reflects the obligatory attention to all parts of a face. The composite face effect was reduced in active stimulation conditions compared with the sham stimulation condition. Additionally, the current polarity did not modulate the effect of tDCS in the two experiments. The present study demonstrates that N170 can be causally manipulated by stimulating the OT with weak currents. Furthermore, our study provides evidence that obligatory attention to all parts of a face can be affected by the commonly used tDCS parameter setting.

## Introduction

Face processing is vital for social life. Therefore, it would be valuable if this ability could be rehabilitated with brain stimulation techniques, especially for those who suffer from face processing deficits. Various studies have adopted event-related repetitive transcranial magnetic stimulation (rTMS) to investigate the function of the right occipital face area (rOFA) [1]–[3] and prefrontal cortex [4], [5] in face processing. Another potential alternative is transcranial direct current stimulation (tDCS). tDCS is a non-invasive brain stimulation technique that applies a weak direct electrical current (usually 1∼2 mA) via the scalp to modulate cortical excitability [6]. tDCS is believed to be able to hyperpolarize (cathode stimulation) or depolarize (anode stimulation) neuronal membranes [7], which assumes anode-excitation and cathode-inhibition effects on brain functions. Recently, beneficial effects of tDCS have been reported in many cognitive domains [8]–[10]. The clinical value of tDCS as a tool in cognitive rehabilitation has also been advocated [9], [11], [12]. Compared with rTMS, tDCS is less expensive and more convenient in the clinical setting. However, the potential of tDCS in modulating high-level perceptual ability, such as face recognition, has been less explored.

Several previous studies have used tDCS to study research questions related with face perception, including emotion recognition from facial expressions [13]–[15], semantic processing in subliminal faces [16], and face identity processing [17]. Among those studies, three chose the prefrontal cortex as the site for stimulation [14], [16], [17], another focused on the temporal lobe [13], and the rest focused on the left occipital lobe [15]. Although the prefrontal cortex, temporal lobe and the left occipital lobe are also relevant in face processing, a more directly related neural network in face processing may be hosted by the occipito-temporal cortex (OT), including several core neural circuits such as the Fusiform Face Area (FFA), the Occipital Face Area (OFA) and the Superior Temporal Sulcus (STS) [18].

Face processing is believed to be dominated by the OT, especially the right OT. Lesion studies have shown that a single lesion in the right OT often causes severe face processing deficits [19]–[26]. Neuroimaging studies in healthy adults also revealed right hemispheric lateralization in the activation level of FFA [27], [28] and N170, a face-sensitive ERP component originating from the OT [29]–[31]. Typically, recognizing inverted faces takes longer and elicits a bigger N170 than upright faces in the population of healthy adults. The face inversion effect is always absent in prosopagnosia patients [32], [33]. The neural mechanism of the face inversion effect on N170 is still controversial. One viewpoint is that inverted faces recruit the same neural mechanism for processing upright faces. Source localization studies using EEG or MEG have shown that N170 elicited from upright faces and inverted faces share the same source [34], [35]. Another view is that processing of inverted faces recruits additional neural mechanisms than those used for upright face processing [36]. The core face network in the OT is considered the neural basis of N170 [34], [37]. Currently, the effect of applying tDCS to the OT on neural correlates of face processing, such as N170, has not been investigated.

Human expertise in face recognition is always attributed to configural processing [38]. Maurer, LeGrand and Mondloch (2002) distinguished three types of configural processing: detection of first-order spatial information that defines a face (two eyes above a nose and mouth), holistic processing and sensitivity to spacing among face features [38]. Holistic processing refers to the gestalt perception of face or obligatory attention to all parts or features of a face [39], [40]. For example, recognizing the identity of a feature is better if the face is presented in the context of the entire face rather than as an isolated face feature [41], [42]. This well-known part-whole effect is considered a demonstration of holistic processing. Holistic processing is also demonstrated by the composite face effect [43], [44]. The composite face effect refers to the phenomenon that participants often make errors and have difficulty in judging whether top halves of two faces are same or not when the two faces have the same top halves but different bottom halves [39]. Therefore, the composite face effect reflects the inability to neglect the distractor part of a face, and this failure of selective attention is what the composite face effect is measuring. The composite face effect can effectively predict individual differences in face recognition ability [45]–[47] and is often regarded as the gold standard measure of holistic processing [48], [49].

**Figure 1:**
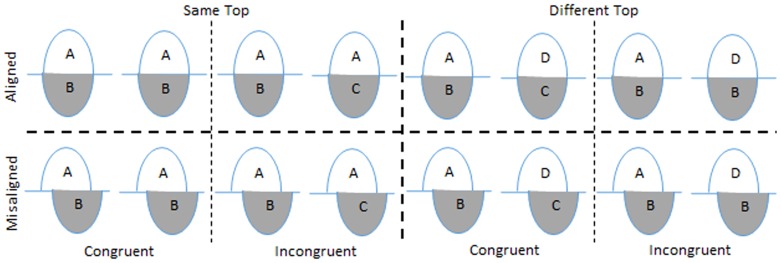
Complete Design of the Composite Face Task. In each face pair, the first composite face is the study face, and the second composite face is the test face. Participants have to attend to the top half shown with a white background and neglect the irrelevant bottom half shown with a gray background. In the congruent condition, the study and the test face halves are either the same or are both different. The task-relevant half (upper) is shown with a white background, and the irrelevant face half (lower) is shown with a gray background. In the congruent condition, the study and test face halves (i.e., cued and irrelevant halves) are either both the same or different. In the incongruent condition, the corresponding top or bottom halves of the study and test faces are the same, and the corresponding irrelevant face halves are different.

There are two variants of the composite face task in the literature: a partial version [50] and a complete version [43], [51]. The complete version can eliminate the confound between the effect of alignment and response bias that may occur in the partial design [45], [52]. The main factors manipulated in the complete version are alignment and congruency (see Fig. 1 for an illustration of the design). Alignment refers to whether the top and bottom half are aligned. Congruency refers to the relationship between the response indicated by the top half (the target) and the response indicated by the bottom half (the distractor). In congruent trials, both top and bottom halves of the two composite faces in a trial are identical or different. In incongruent trials, top halves of the two composite faces are identical but their bottom halves are different, or vice versa. People are prone to make more errors in incongruent trials than congruent trials. The congruency effect therefore reflects the obligatory attention to both halves of the composite faces even if one half is task irrelevant. The misalignment disrupts the configuration of a face. Therefore the congruency effect in the misaligned condition serves as a baseline that reflects the general selective attention to task-irrelevant parts. Typically, the congruency effect is minimized in the misaligned condition. The interaction between alignment and congruency is therefore used to quantify the composite face effect by subtracting congruency effects in the misaligned condition from congruency effects in the aligned condition. A higher value indicates more holistic processing. This index has been shown to be particularly sensitive to holistic processing of faces or familiar objects [53], [54] and can predict people's ability in face recognition [45]. Therefore, several studies have used the interaction between alignment and congruency to define degrees of holistic processing [45], [47], [55], [56]. Previous neuroimaging studies [57], [58] and neuropsychological studies [25], [26] have indicated that OT may play a central role in holistic processing. However, no study has investigated the effect of stimulating the OT with tDCS on the composite face effect, the well-accepted behavioral measure of holistic processing.

The aims of the present study are two-fold: to explore whether stimulating the OT with tDCS can modulate the N170 and holistic processing. In Experiment 1, the effect of tDCS on N170 evoked by faces was investigated. A simple face orientation judgment task was adopted to evoke N170. As previous source localization studies have shown that the OT is the source of N170 [34], [37], we expected that N170 elicited by faces should be modulated if the OT is stimulated with tDCS. Additionally, whether tDCS over the OT modulates upright faces and inverted faces in the same manner is also an interest of the study. In Experiment 2, the effect of tDCS on composite face effect was investigated. The composite face task was used to measure holistic processing.

According to the anode-excitation and cathode-inhibition assumption, tDCS may have the current polarity dependent effect. However, the current polarity effect is not consistently found in the literature [10]. To investigate this assumption in our task setting, the current polarity was also manipulated in the two experiments.

## Experiment 1: The tDCS Effect on N170

### Methods

#### Ethics Statement

The procedure was approved by the Human Research Ethics Committee of the University of Science and Technology of China (USTC) according to the principles expressed in the Declaration of Helsinki. Participants were adult undergraduate students or postgraduate students at USTC. Informed consent was obtained in written form from all participants. The ethics statement applied to the two experiments in the study.

An unexpected issue happened during the implementation of this study. The age of 3 female undergraduates was under 18 by the time they attended the Experiment (two in Experiment 1 (age 16 and 17) and one in Experiment 2 (age: 16)), due to the failure of our experimenters to emphasize the age requirement to the participants and to check the age information in the completed consent form. We contacted with them to evaluate whether they felt any suspicious changes related with attending our study after we sensed this issue. They believed that no side effects were observed due to this study. Additionally, we reported this issue to the Human Research Ethics Committee of USTC. They checked and confirmed that we had explicitly stated the age requirement in the proposed protocol and consent form. They confirmed from our previous follow-up that those three minors did not suffer from any harm because of our study. Because the three participants do not meet the age requirement in our proposed protocol, they were not included in the study sample.

#### Participants

Twenty-seven healthy adult volunteers participated in three sessions of this study. Participants reported no history of chronic neurological, psychiatric, or medical conditions, as well as no current use of psychoactive medication. Participants were also warned to not drink alcohol 24 hours prior to the experiment, and to not drink tea, coffee, or caffeinated drinks 2 hours before each experimental session. Three participants were excluded from analysis because of excessive artifacts in one of the EEG sessions, leaving 24 participants in the final analysis (11 females; mean age  = 22.8±2.3 years, range  = 20 to 31). All participants were right handed and had corrected to normal vision. Informed written consent was obtained prior to study participation. Monetary compensation was provided after the completion of 3 experimental sessions.

#### tDCS protocol

A symmetric bilateral tDCS protocol was used; two tDCS electrodes were positioned on the left and the right OT accordingly. By switching the position of the anode electrode and the cathode electrode, the direction of current flow was manipulated. The stimulation was delivered through a battery-driven stimulator (DC-Stimulator Plus, neuroConn GmbH). Electrodes were inserted into 5×7 cm^2^ saline-soaked synthetic sponges. The P7/P8 are on the border between the temporal and the occipital lobes [59]. Therefore, P7/P8 were chosen as the major sites for stimulation. In the Left-anode-Right-cathode (LaRc) session, the anode electrode was centered over the P7, and the cathode electrode was centered over the P8. The positions of the anode and cathode electrodes were switched in the Left-cathode-Right-anode (LcRa) session. A direct current of 1.5 mA was delivered for 15 minutes in both LaRc and LcRa sessions. The current increased or decreased in a ramp-like fashion over 30 seconds at both the beginning and end of the stimulation. In the sham session, the current increased to 1.5 mA and then decreased to 0 mA within 1 minute.

#### Design and Procedure

We used a single-blind and sham-controlled design. Participants attended 3 tDCS sessions (the LaRc, LcRa, and sham session) that were separated by at least 3 days. The order of the tDCS sessions was counterbalanced between participants. With the help of an EEG quick cap, the saline-soaked electrodes were placed on the scalp with P7 or P8 as the center. Stimulation lasted 15 minutes, during which participants were instructed to close their eyes and relax. Then, tDCS electrodes were removed and the EEG montage was configured in approximately 10 minutes. Participants performed a face orientation judgment task while the EEG was recorded. The task included 16 practice trials and 360 test trials. Sixty unfamiliar upright face images with neutral expression in test trials were adopted from a previous study [41]. Inverted face sets were made by rotating the faces by 180°. A 200 ms fixation indicated the start of a new trial. After a blank screen with a random duration ranging from 100 to 300 ms, a face image (height × width: 5.06°×4.02°) appeared for 200 ms. Then, participants had to press the corresponding key (“z” or “m”) to indicate whether the face was in the upright or inverted direction as quickly as possible within a random time window ranging from 1500 to 2500 ms. The key mapping was counterbalanced between participants. Stimuli were presented on a 21-inch LCD monitor (60 Hz, 1024×768 pixels), which was placed 120 cm from the participant. The presentation was administered using MATLAB (The MathWorks, Inc.) and PsychToolBox [60].

#### EEG recording and data analysis

The EEG was continuously recorded (band-pass filter: 0.05∼100 Hz, sampling rate: 500 Hz) from the scalp using a NuAmp2 amplifier and 64 Ag/AgCl channels mounted in a quick cap and 2 channels at the left and right mastoids. All 66 channels were referenced to the tip of the nose with the ground electrode at the center of AFz and FPz. Horizontal electrooculography (EOG) was recorded using bipolar channels placed lateral to the outer canthi of the two eyes, and vertical EOG was recorded using bipolar channels placed above and below the left eye. Impedance was kept below 10 KΩ. EEG data were analyzed using SCAN 4.3 (NeuroScan Inc.). Ocular artifacts were corrected with a regression-based procedure [61]. Then, the EEG was low-pass filtered using a finite impulse response filter (30 Hz, 24 dB/oct). Separate EEG epochs of 600 ms were segmented and baseline corrected with a 100 ms pre-stimulus onset. With the exception of EOG channels, epochs with voltage exceeding 75 µv at any time for any channel were defined as artifacts and rejected. Finally, epochs were averaged for various experimental conditions, specifically upright faces vs. inverted faces, and re-referenced to a common average reference.

**Figure 2:**
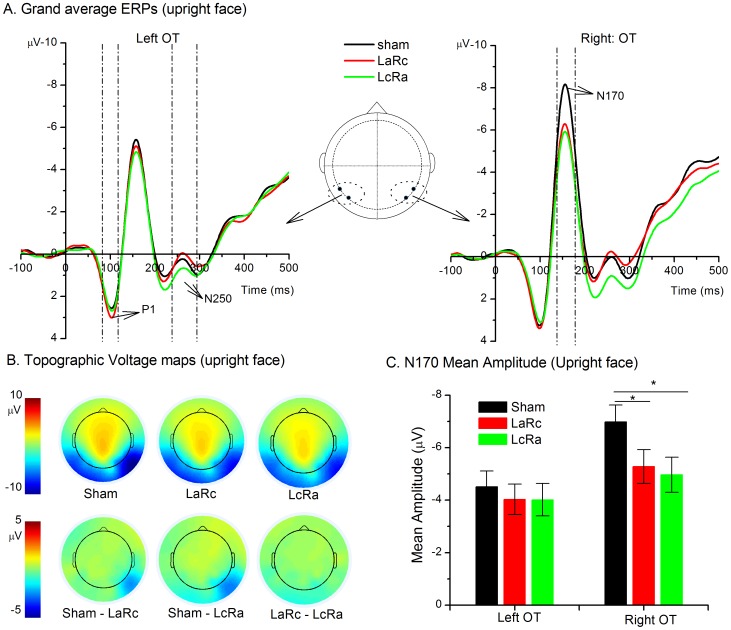
ERPs for the 3 tDCS conditions. (A) Grand average ERPs elicited by faces in 3 tDCS conditions on the left (average of P7 and PO7) and right OT (average of P8 and PO8). The gray bars show the time window for computing the mean amplitude N170 (140-180 ms). (B) Topographic voltage maps showing the spatial distribution of the N170 (averaged over 140 to 180 ms time window) for the sham, LaRc and LcRa condition (first row) and difference waves of N170 (averaged over 140 to 180 ms time window) among the three conditions (second row). (C) The differential effect of tDCS on left and right N170. Error bars represent standard error of mean. * indicates p<.05.

The N170 was quantified using mean amplitudes (140–180 ms) relative to a pre-stimulus baseline of 100 ms at the channels of P7/8 and PO7/8. The time window for computing the mean amplitude was determined by inspecting grand mean waveforms. Consistent with previously reported studies [36], [62], the P7/8 and PO7/8 channels were chosen for further statistical analyses. Furthermore, the N170 component was maximal at P7/8 and PO7/8 consistently across participants in our experiment. In addition to the N170, the P1 and the N250 were also analyzed as they are also related to face processing. The P1 is a positive deflection starting from 60–90 ms after stimuli onset and peaks at approximately 100–130 ms. The P1 has been shown to reflect processing of low-level visual features, which may be immune to top-down influences [63]. The N250 is an ERP component that follows the peak of N170 at approximately 230–300 ms in the OT region, which may index the encoding of face identity [64], [65]. The mean amplitude of P1 (80–120 ms) and N250 (230–290 ms) were also measured from the same channels as the N170 (See Fig. 2A for illustration of the time window of ERPs). The mean amplitude of each component at the P7/PO7 and mean amplitude of each component at the P8/PO8 were averaged to represent the neural activity of the left and right OT respectively. In addition to the mean amplitude, the peak latency of P1 and N170 was also measured and analyzed. Peak latency of N250 was not used because N250 was a sustained potential, and therefore, the peak latency was a poor measure of its activity. To explore the face inversion effect, a repeated measures ANOVA with three within-subject factors, specifically hemisphere (left vs. right), tDCS (sham, LaRc vs. LcRa), and orientation (upright vs. inverted), was performed on the mean amplitude or peak latency of each component separately. To investigate whether N170 elicited by faces was modulated by tDCS, we performed a two-way ANOVA with hemisphere (left vs. right) and tDCS (sham, LaRc vs. LcRa) as factors on upright trials only. The upright face trials and inverted trials were not collapsed in the present study to measure N170 because inverted faces might recruit additional mechanisms [36].

### Results

#### Behavioral Performance

The orientation judgment task was an easy task. Participants performed this task with high accuracy (*M* = 97.7%, *SEM* = 0.4%). A two-way ANOVA on accuracy with tDCS and orientation as within-subject factors yielded no significant effects (tDCS: *F*(2, 46) = .263, *p* = .770; orientation: *F*(1, 23) = 3.151, *p* = .089; tDCS×orientation: *F*(2, 46) = .174, *p* = .841). The same ANOVA procedure performed on mean response times revealed a significant main effect of orientation (*F*(1, 23) = 5.422, *p* = .029), suggesting that responses were faster in upright face trials (*M* = 445 ms, *SEM* = 14.8 ms) as opposed to responses in inverted face trials (*M* = 455 ms, *SEM* = 13.3 ms). Neither the main effect of tDCS (*F*(2, 46) = .363, *p* = .698) nor the interaction between tDCS and orientation (*F*(2, 46) = .298, *p* = .51) was significant.

#### P1

Mean amplitudes of P1, N170, and N250 in various conditions are listed in Table 1 (see S1 Fig. for an illustration of the ERPs in each separate conditions). The 3-way repeated measures ANOVA with factors of hemisphere, tDCS and orientation on the mean amplitude of P1 only revealed a significant main effect of orientation (*F*(1, 23) = 15.658, *p* = .001). The P1 amplitude was enhanced for inverted faces compared with upright faces. None of the other effects reached significance (tDCS: *F*(2, 46) = .1.170, *p* = .319; Hemisphere: *F*(1, 23) = 1.419, *p* = .246; Hemisphere×tDCS: *F*(2, 46) = .102, *p* = .904; Orientation×tDCS: *F*(2, 46) = .624, *p* = .540; Orientation×Hemisphere: *F*(1, 23) = .092, *p* = .765; Orientation×tDCS×Hemisphere: *F*(2, 46) = .138, *p* = .871).

**Table 1:** Mean Amplitude (µV) for P1, N170 and N250 in Experiment 1.

		Left (P7/PO7)			Right (P8/PO8)		
		LaRc	LcRa	Sham	LaRc	LcRa	Sham
Inverted face	P1	2.58±0.38	2.23±0.34	2.34±0.34	2.92±0.43	2.69±0.37	2.75±0.37
	N170	−4.63±0.69	−4.35±0.67	4.85±0.63	−6.01±0.67	−5.73±0.76	−7.65±0.72
	N250	−0.01±0.59	0.63±0.47	0.20±0.49	−0.24±0.65	0.91±0.59	−0.10±0.59
Upright face	P1	2.35±0.36	2.11±0.37	2.06±0.36	2.63±0.43	2.52±0.40	2.48±0.37
	N170	−4.30±0.57	−4.11±0.61	4.51±0.58	−5.28±0.63	−4.97±0.66	−6.98±0.63
	N250	0.34±0.52	0.90±0.51	0.53±0.55	0.15±0.58	1.31±0.58	0.39±0.56

A similar analysis was also performed on the P1 latency. The 3-way repeated measures ANOVA with factors of hemisphere, tDCS, and orientation only yielded a significant main effect of hemisphere (*F*(1, 23) = 9.479, *p* = .005), indicating a delayed P1 latency in the left hemisphere (*M* = 104 *ms*, *SEM* = 1.7) compared with the right hemisphere (*M* = 100 *ms*, *SEM* = 1.9). None of the other effects were significant (all *ps*>.1).

#### N170

The 3-way repeated measures ANOVA with factors of hemisphere, tDCS, and orientation on the N170 amplitude yielded a significant main effect of orientation (*F*(1, 23) = 7.167, *p* = .013), indicating enhanced N170 activity in inverted face trials compared with upright face trials. This inversion effect on the N170 is consistent with previous studies [36]. A general right hemispheric lateralization of the N170 was manifested by a significant main effect of hemisphere, *F*(1, 23) = 13.580, *p* = .001. The main effect of tDCS was also significant, *F*(2, 46) = 4.500, *p* = .016. However, the interaction between tDCS and hemisphere was also significant, *F*(2, 46) = 4.170, *p* = .022, suggesting that tDCS may modulate left and right N170 in a different manner. Other interaction terms involved orientation were not significant, indicating that tDCS did not modulate the N170 inversion effect (Orientation×tDCS×Hemisphere: *F*(2, 46) = .322, *p* = .727; Orientation×Hemisphere: *F*(1, 23) = 3.257, *p* = .084).

To investigate whether tDCS modulates N170 elicited by faces, we performed a two-way ANOVA on N170 in upright face trials. The main effect of tDCS (*F*(2, 46) = 4.817, *p* = .013) and the main effect of hemisphere (*F*(1, 23) = 12.642, *p* = .002) were both significant. Moreover, the interaction between tDCS and hemisphere was also significant (*F*(2, 46) = 4.574, *p* = .015), suggesting differential effects of tDCS on the left N170 and the right N170. For the left N170, the main effect of tDCS was not significant, *F*(2, 46) = .601, *p* = .553. For the right N170, the main effect of tDCS was significant, *F*(2, 46) = 6.230, *p* = .004. Pairwise comparison among 3 tDCS conditions were then conducted using Bonferroni adjusted alpha level of.017 per test (.05/3) (2-tailed). N170 amplitude in both the LaRc and LcRa condition was significantly reduced compared with the sham condition (LaRc vs. sham: *p* = .015; LcRa vs. sham: *p* = .006). No significant difference between LaRc and LcRa condition was found (LaRc vs. LcRa: *p* = .546). See Fig. 2 for the illustration of the N170 results for upright face trials.

Although the main interest of the present study is the N170 elicited by upright faces, we did a similar analysis for inverted face trials. The main effect of tDCS (*F*(2, 46) = 4.002, *p* = .025) and the main effect of hemisphere (*F*(1, 23) = 13.100, *p* = .001) were both significant. Additionally, the interaction between tDCS and hemisphere was also significant (*F*(2, 46) = 3.491, *p* = .039). For the left N170, the main effect of tDCS was significant, *F*(2, 46) = .896, *p* = .415. For the right N170, the main effect of tDCS was also significant, *F*(2, 46) = 4.745, *p* = .013. Multiple comparisons were then conducted using Bonferroni adjusted alpha level of.017 per test (.05/3) (2-tailed) on the right N170. N170 amplitude in both the LaRc and LcRa conditions was reduced compared with the sham condition, which was marginally significant (LaRc vs. sham: *p* = .024; LcRa vs. sham: *p* = .026). No significant difference between LaRc and LcRa conditions was found (LaRc vs. LcRa: *p* = .588).

A similar analysis was also performed on N170 latency. The 3-way repeated measures ANOVA with factors of hemisphere, tDCS, and orientation yielded a significant main effect of orientation (*F*(1, 23) = 73.748, *p*<.001), indicating a delayed N170 in inverted face trials (*M* = 164 *ms*, *SEM* = 1.95) compared with upright face trials (*M* = 158 *ms*, *SEM* = 1.88). None of the other effects were significant (all *ps*>.2).

#### N250

The 3-way repeated measures ANOVA with factors of hemisphere, tDCS, and orientation on the mean amplitude of N250 only yielded a significant main effect of orientation, *F*(1, 23) = 5.574, *p* = .027. Inverted faces elicited more negative N250 compared with upright faces. The main effect of tDCS was not significant, *F*(2, 46) = 2.958, *p* = .062. Additionally, none of other effects reached the significance level (Hemisphere: *F*(1, 23) = .005, *p* = .944; Hemisphere×tDCS: *F*(2, 46) = 1.688, *p* = .196; Orientation×tDCS: *F*(2, 46) = .150, *p* = .861; Orientation×Hemisphere: *F*(1, 23) = .328, *p* = .573; Orientation×tDCS×Hemisphere: *F*(2, 46) = .168, *p* = .846).

In summary, the typical inversion effect was manifested on response times, P1 amplitude, N170 latency and amplitude and N250 amplitude. However, none of those inversion effect could be modulated by tDCS over OT. An interesting finding is that the N170 amplitude was modulated by tDCS; both LaRc and LcRa stimulation reduced the amplitude of N170 for both upright faces and inverted faces.

## Experiment 2: The tDCS Effect on Holistic Face Processing

### Methods

#### Participants

Forty-six healthy volunteers participated in this experiment. All participants were recruited with the same inclusion criteria as Experiment 1. One participant failed to complete the study. Six participants were excluded from data analysis because they did not show any holistic effect in the sham condition. The sample in the final analysis consisted of 39 participants (21 female; mean age  = 22.7±1.9 years, range  = 19 to 26). Informed written consent was obtained at the beginning of the first session and monetary compensation was given after completing 3 experimental sessions.

#### tDCS protocol

There were 3 tDCS sessions (LaRc, LcRa and sham). The tDCS setting was the same as Experiment 1.

#### Design and procedure

The experiment was single blind and sham controlled. Participants were measured using the composite face task in three sessions during online tDCS stimulation, separated by at least 72 hours. The order of tDCS sessions was counterbalanced between participants. In each session, participants were first configured with tDCS electrodes. After the start of tDCS stimulation, participants performed the composite face task. The task included a practice part (approximately 3 minutes) and a test part (approximately 12 minutes).

The stimuli used in the task were composite faces made by combining the top half of a face and the bottom half of another face. Images of 5 male faces were adopted from a previous study [46]. A set of composite faces was created by randomly combining a top half of a face with a bottom half of another face. Single trial events in the composite face task were structured in the following manner. A fixation of 250 ms indicated the start of a new trial. Then, the first composite face was presented for 200 ms followed by a blank screen lasting 800 ms. Finally, the second composite face appeared for another 200 ms. Participants indicated whether the top halves of the two composite faces were the same or different by pressing “z” or “m”, respectively, with no time limitation. The accuracy instead of response times was emphasized. The response-key mapping was counterbalanced between participants. The face stimuli were approximately 5.59°×3.96° (height × width) in aligned trials and 5.59°×5.58° (height × width) in misaligned trials. A different set of face stimuli was used in practice trials. Stimuli were presented on a 17-inch CRT monitor (75 Hz, 1024×768 pixels), which was placed at 48 cm from the participant by using a chin rest. The presentation was administered using MATLAB (The MathWorks, Inc.) and PsychToolBox [60].

The design of this task included two important factors: congruency and alignment. Congruency refers to the relationship between the response indicated by the top half (the target) and the response indicated by the bottom half (the distractor). Another manipulation was alignment, which means whether the top and bottom part were aligned with each other. The task used in this experiment had 32 practice trials and 160 test trials (40 for each alignment×congruency condition).

#### Data analysis

Signal detection theory measure of sensitivity (A′) was used as the dependent measure [66]. A′ is a nonparametric measure of sensitivity which can be relatively robust compared with d′ if the assumptions of normality and equal variances are violated [67]. A′ is computed with the following formula: A′ = 0.5+*sign*(H − F)×[(H − F)^2^+|H − F|]/[4×max(H, F)−4×H×F]. H and F represent hit rate and false alarm rate, respectively. The *sign* function is used to extract the sign (“+” or “−”) of a number. A three-way repeated ANOVA with tDCS, alignment and congruency as within-subject factors was performed on A′. A three-way interaction between tDCS, alignment, and congruency was expected. The holistic processing was indexed by the interaction between alignment and congruency, computed with the function [(A′_aligned-congruent_ – A′_aligned-incongruent_) - (A′_Misaligned-congruent_ – A′_ Misaligned-incongruent_)].

### Results

#### Holistic Effect

**Figure 3:**
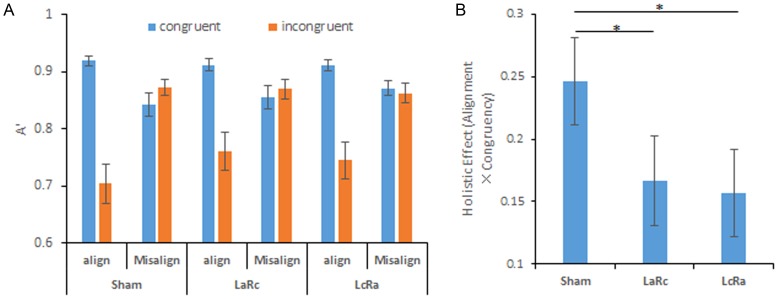
Behavioral results of Experiment 2. (A) A′ for each tDCS × Alignment × Congruency conditions. (B) Holistic effect in each tDCS condition. Error bars represent standard error of mean. * indicates p<.05.

A three-way 2 (Alignment: align vs. misalignment) ×2 (Congruency: congruent vs. incongruent) ×3 (tDCS: sham, LaRc, vs. LcRa) ANOVA was performed on A′. The general performance was the same in all three tDCS sessions, according to the non-significant main effect of tDCS (*F*(2,76) = 1.074, *p* = .347). The main effect of alignment was significant (*F*(1,38) = 11.333, *p* = .002), suggesting performance in misaligned trials (*M* = 0.862, *SEM* = .014) was better than the aligned trials (*M* = 0.825, *SEM* = .017). The main effect of congruency was also significant (*F*(1,38) = 33.430, *p*<.001). Performance in congruent trials (*M* = 0.885, *SEM* = .012) was better than incongruent trials (*M* = 0.803, *SEM* = .020). The interaction between alignment and congruency was also significant (*F*(1, 38) = 37.007, *p*<.001), suggesting a significant overall holistic effect, specifically a larger congruency effect in aligned trials compared with misaligned trials. Importantly, the three-way interaction between tDCS, alignment and congruency was also significant (*F*(2, 76) = 5.833, *p* = .004). The composite face effect was computed by subtracting the congruency effect in misaligned trials from the congruency effect in the aligned trials. A one way AVOVA with tDCS condition as a within subject factor was performed on the composite face effect. The main effect of tDCS was significant, *F*(2, 76) = 5.833, *p* = .004. Pairwise comparison among 3 tDCS conditions were then conducted using Bonferroni adjusted alpha level of.017 per test (.05/3) (2-tailed). Composite face effect in both LaRc and LcRa conditions was significantly reduced compared with the sham condition (LaRc vs. sham: *p* = .011; LcRa vs. sham: *p* = .003). No significant difference between LaRc and LcRa condition was found (LaRc vs. LcRa: *p* = .736) (see Fig. 3B for an illustration).

### General Discussion

Transcranial direct current stimulation is a promising technique in cognitive rehabilitation. However, limited research efforts have been made in investigating the effect of tDCS on face processing, a vital perceptual ability for social well-being. The OT has been associated with face perception. The present study investigated whether stimulating the OT with tDCS can affect face processing. Experiment 1 demonstrated that active tDCS stimulation over the OT reduced the N170 amplitude in the right hemisphere compared with sham stimulation, and the stimulation effect was independent of the current polarity. However, the inversion effect on both behavioral measures (response times) and ERPs measures (P1, N170 and N250) were not modulated by tDCS. Experiment 2 further revealed that composite face effect, a behavioral measure of holistic processing, was attenuated in active stimulation compared with sham stimulation. Consistent with Experiment 1, no current polarity dependent effect was found.

Discriminating whether a face is upright or inverted is a simple task that may only depend on some basic process, such as extracting a typical “T” structure (two eyes above nose and mouth) that defines a face [38]. This process may be robust and therefore inflexible to external interference. Only some powerful factors, such as aging [68] and lesion [33], have been reported to change the pattern of face inversion effect. The commonly adopted tDCS parameter setting may be too weak to penetrate this basic process. Holistic processing which reflects the obligatory attention to all parts of a face, might be a flexible process. For example, the composite face effect can be modulated by priming the global or local Navon stimuli [69]. The composite face effect was modulated by tDCS in our Experiment 2. However, it should be noted that the composite face effect was attenuated in degree instead of completely abolished as the case in prosopagnosia patients [48]. Our results imply that the commonly used tDCS dose is enough to modulate face perception at the attention level.

Although neuroimaging studies have revealed that neural circuits in the OT serve as a “core system” in face processing [18], those studies are correlational in nature. Lesion studies on patients serve as a strong test about the casual relationship between OT and normal face perception. However, the population of lesion patients may differ greatly from a healthy adult population, which may inhibit the generalization of findings from the patient population to the healthy adult population. Non-invasive brain stimulation techniques, such as TMS or tDCS, can help researchers directly manipulate neural circuits to reveal the behavioral consequences in healthy adults. Previous studies using rTMS have demonstrated that rOFA, an important part of OT, is critical in many aspects of face processing, such as early analysis of face features [70], gender and trustworthiness judgment [2]. The present study extended this area of research by showing that N170 elicited by face and the composite face effect can be causally manipulated by applying weak direct currents over the scalp of the OT region.

An interesting issue in the present study is the tDCS effects on N170 and composite face effect was very similar. Both LaRc stimulation and LcRa stimulation reduced the N170 and composite face effect compared with sham stimulation. N170 has been associated with the structure encoding stage, in which the gestalt of holistic representation was extracted from faces [29], [71]. Therefore, the coincidence between tDCS effect on N170 and composite effect in the present study might indicate a correlation between N170 and holistic processing. However, because the N170 and composite face effect is not collected within a single experiment, a direct link cannot be established with the current study. In future studies, an individual difference approach to directly investigate whether N170 can be correlated with behavioral hallmarks, such as composite face effect and part-whole effect, should be adopted.

The anode-excitation and cathode-inhibition assumption predicts the current polarity dependent effect. However, LaRc and LcRa stimulation did not differ in both our Experiment 1 and 2. The null effect of current polarity has also been reported by additional studies that used bilateral tDCS stimulation on the DLPFC [72]–[75]. The anode-excitation and cathode-inhibition assumption was based on the logic that tDCS modifies the local cortical excitability underlying tDCS electrodes. However, this notion does not fully explain the tDCS effect, as several studies have demonstrated that not only the local activity but also the connectivity within a distributed neural network was modulated by tDCS [76], [77]. Moreover, face processing depends on separate neural circuits (such as OFA and FFA) as well as the connectivity between them [78]. A possible explanation for our results is that face processing may depend on a balance between the left and right hemispheres (e.g., the connectivity between the left OFA/FFA and the right OFA/FFA) that were modified by applying current stimulation over bilateral OT. Another possible explanation is that both anode stimulation and cathode stimulation to the right OT only modified the default connectivity within the right OT whereas the left hemisphere was unaffected at least in the N170 time window. For example, only the connectivity between the right OFA and the right FFA was modulated. A recent EEG-fMRI fusion study has suggested that a larger N170 amplitude is associated with a stronger connectivity between the right OFA and the right STS [79]. In Experiment 1, only the right N170 was modulated by the tDCS, and the left N170 was not affected. Therefore, the bilateral tDCS setting may modify the default mode of connectivity within the right hemisphere only. However, it is impossible to tease apart the two hypotheses given the current data. To investigate this issue, using fMRI in future studies to systematically compare unilateral and bilateral tDCS stimulation to the OT would be valuable.

The clinical potential of tDCS in improving cognitive abilities has been advocated widely. Several studies have reported the positive evidence of tDCS in enhancing visual abilities, such as attention [80] and change detection [81]. However, the potential of tDCS in modulating high level perceptual ability, such as face, object, and word recognition has been less explored. The effect of tDCS on face processing is not consistently reported in the literature. Additionally, the task context varied among studies. For example, the modulatory effects of stimulating the temporal lobe on a facial expression go/no-go task were different for males and females [14]. Emotional face identification performance was subtly enhanced by anode stimulation to the prefrontal cortex [13]. The subliminal semantic priming by face identity was abolished by cathode stimulation to the left DLPFC [16]. Among those, we are the first to investigate the effect of stimulating the OT on two important basic types of configural processing. Future studies should vary the tDCS parameters and electrode positioning to fully explore the potential of tDCS in cognitive rehabilitation of perceptual abilities.

A potential limitation of Experiment 2 is using the same set of composite faces in all three sessions. To minimize the familiarity effect, the intervals between the 3 sessions were set at least for 72 hours in the present study. Our results showed that the general accuracy among the three tDCS sessions did not differ. Moreover, the validity of the composite face paradigm is not affected by familiarity. The typical composite face effect is found for unfamiliar faces [39], [45] as well as familiar faces [82], [83]. Another limitation is that the underlying neural mechanism of tDCS effect in our study is still not conclusive. Computational models of current flow have indicated that conventional tDCS methodology using two large electrodes positioned on the head disperse current through much of the cortex and even deep brain structures [84]. Some fMRI studies have also shown that tDCS not only modulates local activity but also the connectivity within a network [76], [85]. Because different components in the core neural system of face processing, such as FFA, STS and OFA, interact with each other, it is impossible to conclude whether local activities of each component of face network are modulated by tDCS or the interaction among those components are affected. It is valuable to use fMRI to clarify the neural mechanism of tDCS effect on face processing.

Although the OT, especially the right OT, has been associated with face processing, at least two studies have shown that the right OT is increasingly sensitive to objects as we become familiarized with them [55], [86]. Given the current study, it is difficult to determine whether stimulating the OT with tDCS affects special face processing [87] or a general process underlies perceptual expertise [53], [55], [88]. An artificial object training paradigm adopted in previous studies [53], [55] can build face-like expertise for novel objects. Whether stimulating the right OT can have a similar effect on processing faces and novel objects that we have been extensively trained to recognize is a valuable research direction.

In summary, the present study demonstrated that applying weak currents over bilateral OT could affect N170 in the right hemisphere and composite face effect. These results support the notion that the OT is the source of N170 and provides evidence that the fundamental process in face perception, such as holistic processing, can be modulated by tDCS. Contrary to the anode-excitation and cathode-inhibition assumption, no current polarity effect was found in our two experiments. Future studies are needed to fully explore the effect of tDCS in face processing, which can inspire translational studies on cognitive rehabilitation of perceptual deficits in face and object perception.

## Supporting Information

S1 Fig **ERPs for each tDCS×orientation condition at the left (P7/PO7) and right OT (P8/PO8).**
(TIF)
