# Correction: Electrical Stimulation over Bilateral Occipito-Temporal Regions Reduces N170 in the Right Hemisphere and the Composite Face Effect

**DOI:** 10.1371/journal.pone.0119249

**Published:** 2015-03-18

**Authors:** The PLOS ONE Staff

The following information is missing from the Funding section: the Natural Science Fund Project of Anhui Province (1208085MH179).
