# Supplementary figures and images for: Electrical Stimulation over Bilateral Occipito-Temporal Regions Reduces N170 in the Right Hemisphere and the Composite Face Effect
Source: PLoS One. 2014 Dec 22;9(12):e115772. doi: 10.1371/journal.pone.0115772 (PMC4274090; doi:10.1371/journal.pone.0115772)

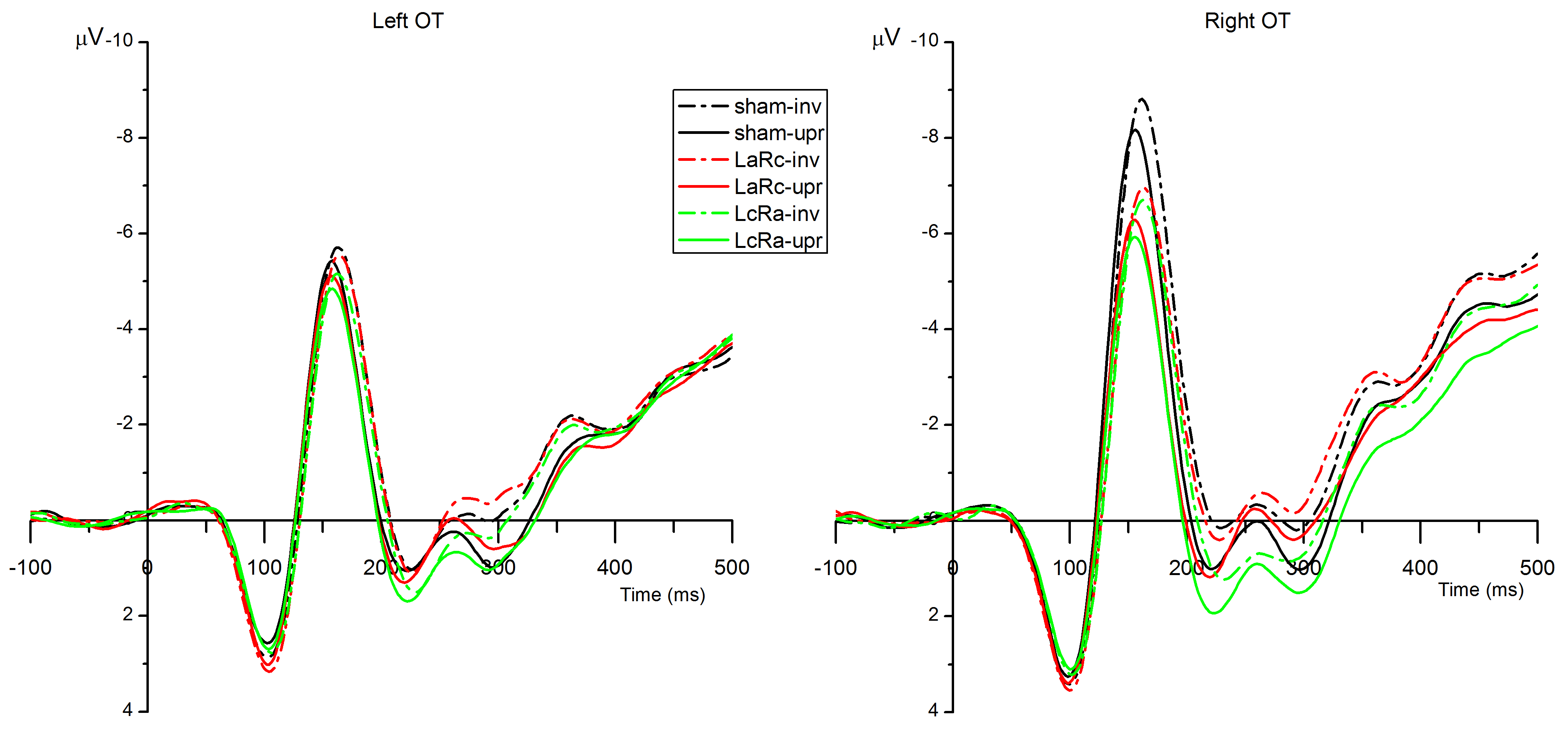

Supplement: S1 Fig — ERPs for each tDCS×orientation condition at the left (P7/PO7) and right OT (P8/PO8). (TIF) [file pone.0115772.s001.tif]
